# Phenytoin‐induced choreoathetosis after serial seizures due to traumatic brain injury and chronic alcoholism

**DOI:** 10.1002/ccr3.1870

**Published:** 2018-10-17

**Authors:** Josef Finsterer, Hans Keller, Alice Reining‐Festa, Barbara Enzelsberger, Franz Weidinger

**Affiliations:** ^1^ Krankenanstalt Rudolfstiftung Vienna Austria; ^2^ 2nd Medical Department with Cardiology and Intensive Care Medicine Krankenanstalt Rudolfstiftung Vienna Austria

**Keywords:** antiepileptic drugs, diphenyl‐hydantoin, epilepsy, movement disorder, side effects

## Abstract

Intravenous phenytoin (PHT) for suspected seizures may trigger severe choreoathetosis. Discontinuation of PHT results in immediate and complete resolution of hyperkinesias. Co‐medication with valproic acid, levetiracetam, tranquilisers, and anesthetics or alcohol presumably do not modify this adverse effect of PHT. Choreoathetosis can be easily misinterpreted as seizures.

## 1 INTRODUCTION

From the literature, it is known that phenytoin (PHT), also known as diphenyl‐hydantoin, occasionally induces reversible hyperkinesias, in particular chorea.^1^, ^2^, ^3^, ^4^ PHT‐induced hyperkinesias may develop under normal as well as elevated serum PHT levels 5, 6 and together with or without cerebral lesions on imaging. Whether simultaneous application of tranquilisers, anesthetics, analgesics, levetiracetam, valproic acid, and elevated serum alcohol levels particularly promote PHT‐induced hyperkinesias is unknown. Here, we report a patient with chronic alcoholism and epilepsy who developed marked hyperkinesias after adding PHT to various other drugs.

## 2 CASE REPORT

The patient is a 70‐year‐old male, height 178 cm, weight 80 kg, who was admitted after a fall of unknown cause, complicated by traumatic brain injury (TBI) with bilateral fronto‐basal contusional bleedings with perifocal edema and blood deposits along the falx and the right tentorium, and a subdural hematoma with subarachnoidal fractions extending along the right fronto‐parieto‐temporal convexity. His previous history was noteworthy for mild paraparesis of the lower limbs since birth being attributed to birth trauma, poliomyelitis, or cerebral palsy, bilateral hip dysplasia since birth, chronic alcoholism, arterial hypertension, inguinal hernia, reflux esophagitis, bougienage of the esophagus at age 66 years because of a cardia stenosis, right‐sided hip total endoprosthesis, and deep venous thrombosis of the right lower limb, complicated by bilateral pulmonary embolism at age 66 years.

Shortly after TBI, the patient developed a series of generalized tonic clonic seizures, complicated by respiratory insufficiency requiring intubation and artificial ventilation (hospital day (hd) 1). As an antiepileptic treatment, levetiracetam (2000 mg/d) and midazolam (7 mg/h) were given. Additionally, he received norepinephrine, clonidine, ketamine, propofol, and sufentanil. Because of suspected increased intracranial pressure (ICP), an ICP probe was implanted from the left frontal side. There was lactic acidosis of 14 mmol/L, which regressed within 24 hours to normal values. Since the serum alcohol level was elevated to 1.92 g/L on admission, intravenous vitamin‐B1 (300 mg/d) and oxazepam were given. On hd3, the patient was transferred to another intensive care unit. Despite rapid reduction of the sedating medication, the patient did not awake. There was megaloblastic anemia, when vitamin‐B12 and folic acid were added. Hypothyroidism with a TSH level of 6.6U/mL (n, 0.2‐3.7U/mL) was appropriately substituted. There was a mild, transient elevation of transaminases and the gamma‐glutamyl‐transpeptidase during hospitalization.

Because of suspected recurrence of seizures on hd5, PHT (1000 mg/d) was administered intravenously. Shortly after starting PHT, spontaneous, overshooting movements of the head, upper limbs, and lower limbs became apparent. Initially, these hyperkinesias were misinterpreted as exacerbation of seizures due to ineffectivity of LEV and PHT, why valproic acid (VPA) was added. Repeated EEG recordings, however, did not show epileptiform discharges. Hyperkinesias occurred daily, either induced by care treatments or spontaneously. They were described as choreoathetotic and occurred in waves every 30‐60 minutes and lasted for about 1 minute. Treatment with tiapride or quetiapine was ineffective. On hd6, the ICP probe was removed since ICP values were recurrently within normal limits. Clinical neurologic exam on hd13 revealed spoor. Strong pain stimuli induced stereotypic flexion contractions of the lower arms and athetoid rolling motions of the head. There was skewed deviation of the bulbs and the Babinski sign was positive bilaterally. VPA was withdrawn without effect on the hyperkinesias.

On neurologic exam at hd15 under remifentanil, clonidine, tiapride, and artificial ventilation, the patient was awake, but did not follow any instructions. There was right‐sided ptosis. Upon passive mobilization, he reacted with repeated stereotypic, choreatic head movements to the right and athetoid flexions of the upper limbs. These hyperkinesias also occurred spontaneously. There was mild rigor bilaterally. Patella tendon reflexes were exaggerated. Since PHT was suspected to be responsible for choreoathetosis, it was immediately discontinued. On hd16, the PHT serum level was still elevated to 118 µmol/L (n, 39.8‐79.2 µmol/L). However, within 48 h after discontinuation of PHT, choreoathetosis completely resolved. Clinical neurologic exam on hd17 confirmed complete resolution of hyperkinesias. On hd19, the patient was tracheostomized. During the further course, the patient awaked and followed simple instructions. Hyperkinesias did not recur. One week after discontinuation of PHT, the serum level of PHT was zero.

## 3 DISCUSSION

From the literature, it is well known that PHT can induce hyperkinesias such as chorea.^1^, ^2^, ^3^, ^6^ Side effects of PHT other than hyperkinesia include gingival hyperplasia,^7^ irreversible hypertrichosis,^7^ encephalopathy,^8^ sedation,^5^ a cerebellar syndrome,^5^ psychosis,^5^ locomotor dysfunction,^5^ megaloblastic anemia,^5^ decreased serum folate levels,^5^ osteoporosis,^5^ liver disease,^5^ IgA deficiency,^5^ a lupus‐like hypersensitivity syndrome,^5^ and lethal Stevens–Johnson syndrome.^7^ The cause of PHT‐induced hyperkinesias is unclear but it has been speculated that it could be a toxic effect^9^ since PTH is well known to be mitochondrion‐toxic.^10^ Some studies suggested that antecedent damage of the basal ganglia could promote the development of PHT‐induced hyperkinesias.^9^ In an animal model of tardive dyskinesia, it has been shown that PHT enhanced the neuroleptic‐induced behavioral hypersensitivity in these animals.^9^ In a 3‐year‐old female with epilepsy and chorea, it was suspected that the interaction between PHT, phenobarbital, and clobazam was responsible for the development of hyperkinesias.^3^ Withdrawal of PHT in this patient resulted in complete resolution of chorea within 24 hours.^3^ Cerebral MRI did not show any lesion of the basal ganglia.^3^ In a 16‐year‐old retarded patient, transient hyperkinesia was observed after a single intravenous infusion of PHT.^6^ This particular patient had experienced spontaneous episodes of hyperkinesias already before application of PHT, why PHT was assumed to have unmasked a pre‐existing latent movement disorder.^6^ In a review of three previously reported cases, PHT‐induced hyperkinesias persisted during 5 years despite normal PHT serum levels.^11^ The authors suspected that PHT induced hyperkinesias by increasing dopaminergic and serotoninergic activity in the basal ganglia^11^ and that patients with pre‐existing basal ganglia damage are susceptible to develop hyperkinesias under PHT.^11^ They found that the frequency of cerebral lesions on imaging in patients with PHT‐induced hyperkinesias is generally high.^11^ The hyperkinesiogenic effect of PHT has been also recognized in other patients.^12^, ^13^

Whether the co‐medication with tiapride, quetiapine, LEV, VPA, tranquilisers, analgesics, and anesthetics triggered or enhanced hyperkinesias, remains speculative. Since hyperkinesias resolved after discontinuation of PHT, a strong influence of other compounds on hyperkinesias is rather unlikely. However, an enhancing effect of any of these substances cannot be definitively excluded. Since VPA does not induce hyperkinesias and was stopped already2 days prior to discontinuation of PHT, it is unlikely that VPA had an enhancing or triggering effect. Furthermore, VPA had no effect on the severity of hyperkinesias. Alcohol most likely did not influence hyperkinesias as the alcohol serum level was presumably zero on hd5 when hyperkinesias were first recognized. LEV had not been reported to trigger hyperkinesias, which is why it is rather unlikely that LEV had an enhancing or triggering effect. On the contrary, LEV is rather beneficial for hyperkinesias as has been shown in patients with choreoathetosis due to cerebral palsy.^14^ Interactions between PHT and the other drugs, however, cannot be excluded to have played a pathogenetic role. Sopor under minimal sedation and vegetative state was attributed to the extensive bifrontal contusional lesions.

In conclusion, this case shows that intravenous PHT for suspected seizures may trigger severe choreoathetosis predominantly of the head and the upper limbs and that discontinuation of PHT results in immediate complete resolution of these hyperkinesias. Co‐medication with VPA, LEV, tranquilisers, analgesics, and anesthetics is rather not responsible for the development of hyperkinesias since they remained absent under these drugs after discontinuation of PHT. A subclinical premorbid condition as a modifier, however, cannot be definitively excluded.

## CONFLICT OF INTEREST

None declared.

## AUTHOR CONTRIBUTION

JF: designed the study, searched for literature, and wrote the first draft. KH: recorded the patients status, searched for literature, critical review. AR‐F: recorded the patients status, searched for literature, critical review. BE: recorded the patients status, searched for literature, critical review. FW: recorded the patients status, searched for literature, critical review.
